# The Present State and Future Perspectives of Cardiac Regenerative Therapy Using Human Pluripotent Stem Cells

**DOI:** 10.3389/fcvm.2021.774389

**Published:** 2021-12-08

**Authors:** Yusuke Soma, Yuika Morita, Yoshikazu Kishino, Hideaki Kanazawa, Keiichi Fukuda, Shugo Tohyama

**Affiliations:** Department of Cardiology, Keio University School of Medicine, Tokyo, Japan

**Keywords:** regenerative therapy, embryonic stem cell (ES cells), induced pluripotent stem cell (iPS cell) (iPSC), stem cell metabolism, heart failure

## Abstract

The number of patients with heart failure (HF) is increasing with aging in our society worldwide. Patients with HF who are resistant to medication and device therapy are candidates for heart transplantation (HT). However, the shortage of donor hearts is a serious issue. As an alternative to HT, cardiac regenerative therapy using human pluripotent stem cells (hPSCs), such as human embryonic stem cells and induced pluripotent stem cells, is expected to be realized. Differentiation of hPSCs into cardiomyocytes (CMs) is facilitated by mimicking normal heart development. To prevent tumorigenesis after transplantation, it is important to eliminate non-CMs, including residual hPSCs, and select only CMs. Among many CM selection systems, metabolic selection based on the differences in metabolism between CMs and non-CMs is favorable in terms of cost and efficacy. Large-scale culture systems have been developed because a large number of hPSC-derived CMs (hPSC-CMs) are required for transplantation in clinical settings. In large animal models, hPSC-CMs transplanted into the myocardium improved cardiac function in a myocardial infarction model. Although post-transplantation arrhythmia and immune rejection remain problems, their mechanisms and solutions are under investigation. In this manner, the problems of cardiac regenerative therapy are being solved individually. Thus, cardiac regenerative therapy with hPSC-CMs is expected to become a safe and effective treatment for HF in the near future. In this review, we describe previous studies related to hPSC-CMs and discuss the future perspectives of cardiac regenerative therapy using hPSC-CMs.

## Introduction

The incidence of heart failure (HF) is high in the aged population, which is growing at a rapid pace worldwide. HF patients with who are resistant to optimal medication and device therapy are candidates for heart transplantation (HT). However, the number of HTs is <100 per year in Japan, and the mean waiting period is ~4 years (1). Cardiac regenerative therapy using human pluripotent stem cells (hPSCs) such as human embryonic stem cells (hESCs) and human induced pluripotent stem cells (hiPSCs) is expected to become an alternative to HT for patients with severe HF (2–4).

**Figure 1:**
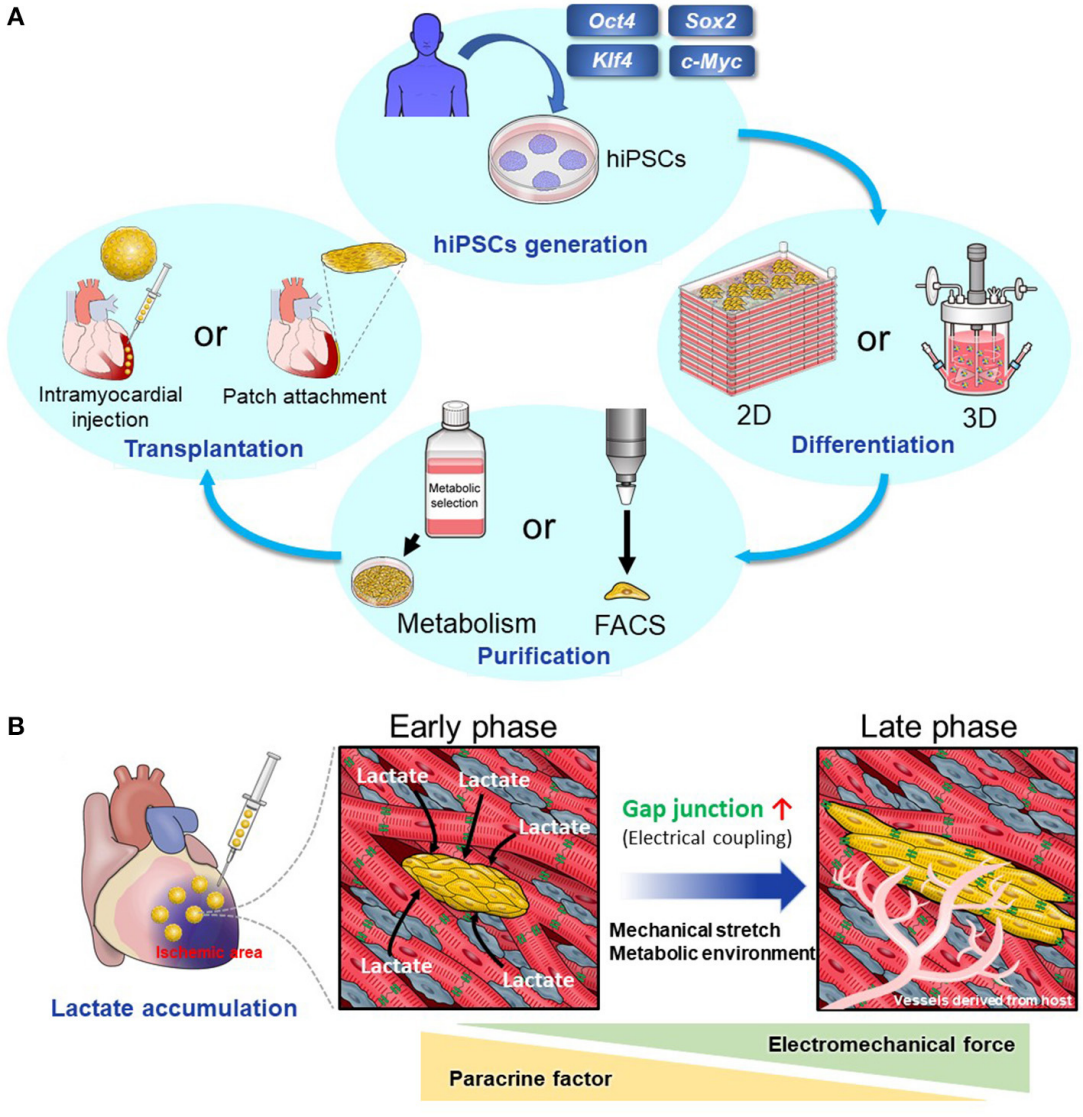
**(A)** The scheme of cardiac regenerative therapy. hPSCs were established by introducing transcription factors including Oct4, Sox2, Klf4, and c-Myc to human somatic cells. Two-dimensional or three-dimensional large-scale culture systems for hPSCs and hPSC-CMs have been developed. Using these systems, we can culture a large number of hPSCs and induce their differentiation into CMs effectively. Elimination of non-CMs including undifferentiated hPSCs is important to prevent tumorigenesis. Particularly, metabolic selection is useful and cost-effective. Then, we transplant hPSC-CMs into the host myocardium. There are two main methods. One method is to transplant hPSC-CMs as a patch onto the host epicardium. The other method is to inject them using a needle into the host myocardium. **(B)** Intramyocardial transplantation of metabolically selected cardiomyocytes. hPSC-CMs that have undergone the metabolic selection in glucose- and glutamine-free medium supplemented with lactate are likely to engraft and become mature when they are transplanted into the ischemic region because of lactate accumulation. There is a high density of host-derived microvessels within the graft, which promotes the engraftment and maturation of transplanted hPSC-CMs.

A large number of hPSC-derived cardiomyocytes (hPSC-CMs) are required for cardiac regenerative therapy (5). Many studies have revealed that differentiation of hPSCs into cardiomyocytes (CMs) can be induced by imitating normal heart development, particularly by controlling developmental signals such as Wnt and BMP signal (6–11). Moreover, in recent years, the following issues have been explored, and many possible solutions have been discovered: (1). Elimination of non-CMs, including undifferentiated hPSCs, and selection of hPSC-CMs to prevent tumorigenesis after transplantation, (2). Large-scale cultures of hPSCs and hPSC-CMs, (3). Effective transplantation of hPSC-CMs into host myocardium (Figure 1A). The following issues remain, and further research is necessary: (1). Arrhythmia after transplantation of hPSC-CMs and (2). Immune rejection. We will discuss these issues and the studies that have been conducted to solve them.

## Elimination of Undifferentiated hPSCs and Selection Of hPSC-CMs

When hPSC-CMs are transplanted into the host myocardium, residual non-CMs, including undifferentiated hPSCs, are a problem because these cells can induce tumors such as teratomas. Even if the contamination of undifferentiated hPSCs spiked into non-tumorigenic cells is ~0.025%, teratomas may be generated if these cells are injected into the legs of immunodeficient mice (12). To solve this problem, methods to eliminate undifferentiated hPSCs or to select only hPSC-CMs have been developed (Table 1A) (13–28).

Table 1Overview of the main studies investigating **(A)** elimination of undifferentiated hPSCs or selection of hPSC-CMs and **(B)** large-scale culture of hPSCs and hPSC-CMs.

**Table:** 

**Approaches**	**Major examples**	**Reference**	**Characteristics**	**Advantages**		**Disadvantages**
**(A) Elimination of undifferentiated hPSCs or selection of hPSC-CMs**						
Cell sorting (FACS/MACS)	TRA-1-60, SSEA-4	(13)	hPSC-specific markers	Simple Accurate	–	Requires cell dissociation Laborious
	SSEA-5	(14)				
	Lectin	(15)				
	SIRPA	(16)	hPSC-CM-specific markers		Selective for hPSC-CMs	
	Mitochondria	(17)	Difference in the number of mitochondria			
Addition of compounds	D-3	(18)	Toxicity in hPSCs via alkaline phosphatases	Does not require cell dissociation Applicable to large-scale culture Rapid		Concern about the adverse effects on hPSC-derived differentiated cells
	Inhibitors of survivin	(19)	Inhibition of hPSC-specific antiapoptotic factor			
	Lectin-toxin fusion protein (rBC2LCN-PE23)	(20)	Combines with only hPSCs			
	Clostridium perfringens enterotoxin (CPE)	(21)	Combines with Claudin-6, hPSC-specific marker			
Metabolic regulation	Glucose and glutamine depletion	(22, 23)	Elimination of non-CMs in glucose- and glutamine-free medium supplemented with lactate	Does not require cell dissociation Applicable to large-scale culture Cost-effective Does not require specific compounds	Selective for hPSC-CMs	Cannot use for other hPSC-derivatives
	Methionine depletion	(24)	Induction of hPSC apoptosis		–	Concern about the effects on hPSC-derived differentiated cells
	PluriSlns	(25)	Inhibition of stearoyl-coA desaturase	Does not requires cell dissociation Applicable to large-scale cutlure Cost-effective	–	
	Orlistat	(26)	Inhibition of fatty acid synthase (FASN) Approved as a treatment for obesity		–	
Others	Glypican-3 (GPC3)	(27)	Pluripotent-state-specific immunogenic antigen HLA-I-restricted GPC3-reactive cytotoxic T lymphocytes	Application to vaccinations and T cell therapy targeting GPC3		Cannot eliminate hPSCs completely
	MicroRNA-302a	(28)	Specifically expressed in hPSCs hPSC elimination system using miR-switch	Application to investigation the dynamics based on intracellular information		Complicated

**Table:** 

**Approaches**	**Major examples**		**Reference**	**Yield and CM purity**	**CM purity**	**Inducer**	**Advantages**	**Disadvantages**
**(B) Large-scale culture of hPSCs and hPSC-CMs**								
Three-dimensional culture	Spinner flask		(29)	1.5–2 × 10^9^ CMs in 1L spinner flask after 25 days of differentiation	>90%	CHIR, IWP4	Does not require cell-adhesive coating proteins Can easily harvest cells from medium	Cells are not evenly exposed to culture medium and reagents
	Stirred bioreactor		(30)	4.0 × 10^7^ CMs in 100 mL stirred bioreactor after 10 days of differentiation	up to 85%	CHIR, IWP2		
			(31)	8.0 × 10^7^ CMs in 100 mL stirred bioreactor after 30 days of differentiation	up to 90%	CHIR, IWP2, SB431542, Pur		
			(32)	4.0 × 10^8^ CMs in 350–500 mL stirred bioreactor after 10 days of differentiation	>90%	CHIR, IWP2 (immediate CHIR-to-IWR transition)		
	Microcarriers	+Rocking platform	(33)	~1.2–1.6 × 10^6^ CMs/mL after 12 days of differentiation	~47–66%	CHIR, IWP2	High yield by microcarriers working as matrix	Requires separation of microcarriers from CMs
		+Spinner flask	(34)	~1.3–1.4 × 10^6^ CMs/mL after 10 days of differentiation and 5 days oflactate purification	~73–83%	CHIR, IWR1, ascorbic acid		
Two-dimensional culture	Stack plates with active gas ventilation		(35)	~1.2 × 10^9^ CMs in 10-layer culture plates after 10–12 days of differentiation	~80% (>97% by metabolic selection)	CHIR, BMP4, IWR1	Stable differentiation and purification for hPSC-CMs	Requires cell-adhesive coating proteins Great time and effort to harvest cells

### Cell Sorting

The methods using fluorescence-activated cell sorting (FACS) or magnetic cell sorting (MACS) are reasonable. Antibodies against pluripotent markers such as TRA-1-60, SSEA-4, and SSEA-5 on hPSCs enable their removal (13, 14). In addition, signal-regulatory protein alpha (SIRPA) is expressed specifically on hPSC-CMs, and an antibody against SIRPA can select hPSC-CMs (16). However, since cardiac regenerative therapy requires a large number of CMs, cell sorting is not favorable because it requires considerable effort and time. Therefore, other methods have been developed.

### Addition of Small-Molecule Compounds

Chemical treatment is a fast and inexpensive method for eliminating undifferentiated hPSCs. For example, the addition of a recombinant lectin-toxin fusion protein that combines with hPSCs eliminates them selectively (20). Additionally, the newly designed phospho-D-peptide, D-3, rapidly induces toxicity in hPSCs via alkaline phosphatases and can eliminate them (18).

### Metabolic Regulation

Metabolic regulation affects cell fate and is a cost-effective method for eliminating undifferentiated hPSCs or selecting hPSC-CMs. Methionine and its metabolite, S-adenosylmethionine (SAM), are key regulators of hPSC maintenance and differentiation. Short-term methionine deprivation results in a rapid decrease in intracellular SAM levels, triggering the activation of p53-p38 signaling, reducing NANOG expression, and poising hPSCs for differentiation. Moreover, prolonged methionine deprivation induces hPSC apoptosis and elimination (24).

Elimination of residual proliferating cells, including undifferentiated hPSCs, is effective in preventing tumorigenesis. We focused on the differences in metabolism between CMs and non-CMs. Glycolysis is enhanced in proliferating cells, including undifferentiated hPSCs, even under sufficient oxygen conditions. They produce energy by glycolysis, and they also produce nucleic acids, amino acids, and lipids, which are required for cell proliferation. In contrast, hPSC-CMs produce energy effectively using the tricarboxylic acid (TCA) cycle and fatty acid oxidation. In addition, unlike hPSCs, hPSC-CMs can use lactate as an energy source. Therefore, we selected only hPSC-CMs by culturing them in glucose-free medium supplemented with lactate (22).

Next, we focused on amino acid metabolism. We discovered that hPSCs use glutamine in addition to glucose in the TCA cycle. Using this characteristic of hPSCs, we selected hPSC-CMs more effectively in glucose- and glutamine-free media supplemented with lactate (23). In addition, this method promotes metabolic maturation of hPSC-CMs (36). This means that the hPSC-CMs surviving in glucose-free medium supplemented with lactate alter their metabolism from glycolysis to lactate oxidation, which is similar to the metabolic change in heart development from fetal to neonatal state (37, 38). hPSC-CMs that have undergone this metabolic selection are likely to engraft and become mature when transplanted into the ischemic region where lactate accumulates (39) (Figure 1B).

Lipid metabolism is also important for hPSCs. An inhibitor of stearoyl-CoA desaturase, the key enzyme in oleic acid biosynthesis, induces endoplasmic reticulum (ER) stress and apoptosis in hPSCs (25). Moreover, we revealed that fatty acid synthesis is important for hPSC survival and Orlistat, which inhibits fatty acid synthase, can safely induce cell death in hPSCs via mitochondria-mediated apoptosis (26).

## Large-Scale Culture of hPSCs and hPSC-CMs

The human left ventricle contains approximately four billion cardiomyocytes (5, 40). It has been reported that ~25% loss of cardiomyocytes due to infarction causes HF (41), and it is necessary to assemble over one billion cardiomyocytes for cardiac regenerative therapy (5, 41). There are mainly two methods available to produce these many hPSC-CMs (Table 1B) (29–35).

### Three-Dimensional Culture

Three-dimensional (3D) suspension culture using a large bioreactor or spinner flask is suitable for the large-scale hPSCs culture. The advantage of 3D culture is that it does not require expensive cell-adhesive coating proteins, such as vitronectin and laminin. Moreover, 3D cultures can be easily harvested from medium. In some studies, microcarriers were used in combination with 3D suspension culture, and were used as a matrix for floating cells (33, 34). This is why many studies focus more on 3D suspension large-scale cultures (29–34). For example, Chen et al. reported that, using 3D suspension culture, they could consistently differentiate hPSCs to >90% CM purity with an average yield of ~1.5–2 x 10^9^ CMs/L using 1 L spinner flasks after 25 days of differentiation. They achieved this by modulating the Wnt pathway and optimizing cell aggregate size, small molecule concentrations, induction timing, and agitation rate (29). Halloin et al. applied stirred bioreactor systems and uninterrupted chemical Wnt pathway control during the early stages of differentiation. They obtained yields of ~1 × 10^6^ CMs/mL, ultimately resulting in the production of ~4.0 × 10^8^ CMs at >90% lineage purity after 10 days of differentiation (32).

### Two-Dimensional Culture

The advantage of 2D culture is that cells are evenly exposed to the culture medium and reagents. Because differentiation of hPSCs into CMs and metabolic selection of non-CMs are more effective in 2D culture than in 3D culture, we established a large-scale 2D culture system by stacking 10 culture plates (35, 42). Under normal culture conditions, the proliferation of hPSCs was not stable because the CO_2_ concentration increased and the O_2_ concentration decreased in the medium. Using an active gas ventilation system with 5% CO_2_, we could produce ~1.2 × 10^9^ CMs at ~80% purity per culture and increase the purity to >97% using metabolic selection (35).

Cell-adhesive coating proteins such as vitronectin and laminin are used for 2D culture instead of feeder cells (43, 44); however, they are expensive and may become an obstacle in clinical settings. It has been reported that artificially synthesized polymer-based coatings are cost-effective for the adhesion of hPSCs (45, 46).

To produce large number of hPSCs more cost-effectively, we found that tryptophan (TRP) plays a key role in the proliferation and maintenance of pluripotency. We produced a large number of hPSCs in TRP-supplemented medium in combination with a large-scale 2D culture system (47).

In conclusion, both 3D and 2D cultures could yield a large number of hPSC-CMs required for clinical application, although each method has its advantages and disadvantages.

## The Method for Transplanting hPSC-CMs

There are two main methods for the transplantation of hPSC-CMs into the host myocardium. One method is to transplant hPSC-CMs as a patch on the surface of the host heart. The other method involves injecting hPSC-CMs into the host myocardium using a needle.

### hPSC-CM Patch Attachment

The advantage of transplanting hPSC-CMs as a patch or sheet is that operators can visually confirm attachment to the host heart. Also, the damage to the host myocardium by patch attachment is less than that by the direct injection method. In contrast, hPSC-CM patches transplanted onto the epicardium tended to drop out in a relatively short time. For example, Kawamura et al. reported that transplantation of hPSC-CM sheets to infarcted porcine hearts improved cardiac performance; however, but after 8 weeks after transplantation, very few hPSC-CMs survived (48). Therefore, the reason for the improvement in cardiac function was thought to be mainly due to paracrine effects of cytokines and growth factors secreted by the sheets rather than the direct contraction force. In previous research, the host heart and hPSC-CM patches on the epicardium did not contract synchronously because the epicardium is an electrical insulator (49). In contrast, Higuchi et al. reported that, using high-flux synchrotron X-ray diffraction, hPSC-CM sheets contracted synchronously with the host heart and suggested that the integration depends on the microenvironment of native cardiac tissue and epicardium (50).

Engineered heart tissue (EHT) made from hPSC-CMs is commonly used in drug screening and disease modeling. In addition, studies using EHT for transplantation have also been reported (51–54). In general, EHT was transplanted onto the epicardium and hPSC-CM patches. For example, Li et al. obtained cardiac tissue-like constructs by cultivating hPSC-CMs on low-thickness aligned nanofibers and transplanted them into infarcted rat hearts (51). The constructs improved left ventricular function and promoted angiogenesis in the peri-ischemic zone 4 weeks after transplantation. Querdel et al. created mesh-structured tissue patches consisting of hPSC-CMs. Transplanting them into infarcted guinea pig hearts improved left cardiac function 4 weeks after transplantation. They created human-scale patches consisting of 4.5 × 10^8^ hPSC-CMs. They transplanted the patches into pig hearts and confirmed their technical feasibility, although they were followed up for only 2 weeks (53).

### Intramyocardial Injection of hPSC-CMs

The other method involves injecting hPSC-CMs using a needle into the host myocardium. Transplantation of hPSC-CMs into the border zone of the ischemic area improved left ventricular function in myocardial infarction model experiments in rodents, monkeys, and swine (10, 55–59). It is considered that the improvement in cardiac function is due to the direct binding between the transplanted hPSC-CMs and host CMs in addition to paracrine effects. In some studies, hPSC-CMs and host CMs showed synchronous electrical coupling and contraction. Moreover, hPSC-CMs transplanted into the host myocardium became mature *in vivo* (55–62). Although they were initially as small as fetal CMs, the transplanted hPSC-CMs gradually increased in size over time. In addition, the sarcomere structure developed in a more favorable way, and isoforms of sarcomere proteins changed the mature types after transplantation. Expression of connexin 43, a gap junction protein, was increased in hPSC-CM grafts. Many factors, such as mechanical stretch, electrical stimulation, and paracrine and endocrine effects of cytokines and hormones, are thought to contribute to the maturation of hPSC-CMs *in vivo*. In addition, hPSC-CMs produce vascular endothelial growth factors and promote angiogenesis (59). Therefore, there was a high density of host-derived microvessels within the graft, which promoted the engraftment and maturation of transplanted hPSC-CMs.

However, even if hPSC-CMs are transplanted into single cells, the engraftment rate is very low (63). This is because transplanted hPSC-CMs flow out through the needle hole by beating the host heart. In addition, they undergo apoptosis because there is no scaffolding. Several methods have been developed to increase engraftment. For example, transplantation of cells with extracellular matrix and bioactive agents such as a growth factor, a cell-permeant peptide, a caspase inhibitor and so on was effective (10, 64, 65). Moreover, we developed a method to transplant hPSC-derived cardiac spheroids (hPSC-CSs) consisting of hPSC-CMs (66). By culturing hPSC-CMs in a microwell plate, we generated hPSC-CSs with a diameter of 150–200 μm, consisting of ~1,000 hPSC-CMs. Transplantation of hPSC-CSs into infarcted rodent hearts significantly improved the rate of engraftment and cardiac function compared to transplantation of hPSC-CMs as a single cell. Moreover, hPSC-CSs also improved left ventricular function and promoted angiogenesis in infarcted swine hearts (59). Furthermore, we developed a special transplant needle (66). This needle has six side holes through which the hPSC-CSs can pass without damage. We could transplant hPSC-CSs without inducing complications such as needle perforation, hemorrhage, or tissue injury.

In recent years, it has been reported that transplanting non-CMs in addition to CMs improved cardiac function to a greater extent than transplantation of CM alone. For example, co-transplantation of hPSC-CMs into the myocardium and a human mesenchymal stem cell-loaded patch onto the epicardium amplified cardiac repair in infarcted rat hearts. It has been suggested that paracrine effects induced by the patch promote angiogenesis and engraftment of hPSC-CMs (67). In addition, co-transplantation of hPSC-derived epicardial cells and hPSC-CMs augmented vascularization, resulting in increased graft size and improved cardiac function. hPSC-derived epicardial cells underwent epithelial-mesenchymal transition to fibroblast-like cells. They then secreted the extracellular matrix, which could contribute to cardiac repair (68). Moreover, Sun et al. reported that co-transplantation of microvessels derived from rat adipose tissue and hPSC-CMs promoted a 6-fold increase in hPSC-CM survival and superior functional recovery in infarcted rat hearts (69). The safety and detailed mechanism by which co-transplantation of CMs and non-CMs augments cardiac repair should be analyzed in future studies.

Whether the transplantation of mature hPSC-CMs is better than that of immature hPSC-CMs remains controversial. Funakoshi et al. created mature, compact ventricular CMs from hPSCs. They compared the transplantation of mature and immature CMs in infarcted rat hearts. The transplanted mature CMs had longer sarcomeres and expressed more connexin 43 than immature CMs 8 weeks after transplantation. Therefore, transplanting mature CMs is expected to mitigate arrhythmias and lead to the development of safer therapies. In contrast, the graft size of immature CMs was larger than that of mature CMs because immature CMs had a stronger ability to proliferate (70).

In almost all studies of hPSC-CM transplantation, experimental animals were followed up for up to 3 months. Therefore, the long-term effects of transplantation remain unknown. Moreover, they used myocardial infarction models because it is difficult to create a dilated cardiomyopathy model. These issues should be addressed when cardiac regenerative therapy is applied in clinical settings. Although the methods of transplanting cells by trans-catheter intracoronary infusion or injection from the endocardial side have been mainly applied for the studies using mononuclear cells derived from bone marrow, these studies did not show clear effectiveness (71, 72). Few studies have described the transplantation of hPSC-CMs using these administration methods.

## Other Issues to Be Solved for Cardiac Regenerative Therapy

### Arrhythmia After Transplantation of hPSC-CMs

One of the problems of transplanting hPSC-CMs into the myocardium is the appearance of arrhythmias, such as ventricular tachycardia (VT) (55–59, 62, 73). In many cases, arrhythmia appeared ~1 month after transplantation and subsided spontaneously. A study using electrical mapping and pacing suggested that the mechanism of VT after transplantation was not macro-reentry but automaticity (62). Initial hPSC-CMs were electrophysiologically immature. For example, the resting membrane potential is less hyperpolarized, and the upstroke velocity of the action potential is much slower in immature hPSC-CMs than in mature ventricular CMs (74). The immaturity of hPSC-CMs, and contamination of pacemaker cells and non-CMs might be responsible for arrhythmia. Nakamura et al. reported that a combination of amiodarone and ivabradine could effectively suppress cardiac death and unstable arrhythmia, although drug treatment does not abrogate arrhythmia completely (73).

### Immune Rejection

Another problem with hPSC-CM transplantation is immune rejection. In the present state, allotransplantation is considered favorable because it is necessary to use hPSCs with proven quality and safety. Immunosuppressants should be used to reduce immune rejection. The optimal regimen of immunosuppressants after transplantation of hPSC-CMs is not yet known, although the regimen for patients who undergo HT is helpful. In addition, we should match the hPSC-major histocompatibility complex (MHC) and recipient-MHC in order to reduce the severity of rejection. This is a concern for patients who do not have MHC-compatible hPSCs. Hypoimmunogenic hPSCs are being developed using genetic modification to enable allotransplantation in patients with multiple types of MHCs. For example, hPSCs that are deficient in HLA-I via disruption of beta 2-microglobulin can evade recognition of recipient T cells, but they can be attacked by NK cells (75). Therefore, it is necessary to develop hypoimmunogenic hPSCs that can evade the recognition of both T and NK cells. For example, hPSCs that are deficient in HLA-I and HLA-II and express CD47 (76), and hPSCs that are deficient in HLA-A and HLA-B and retain HLA-C (77), can evade the attack of both T cells and NK cells. Because these studies used CRISPR/Cas9 technology, the safety of hPSCs that are genetically modified using this technology should be evaluated.

## Future Perspectives of Cardiac Regenerative Therapy

Many problems are gradually being solved to achieve cardiac regenerative therapy. Menasché et al. reported that they transplanted ~8.2 million hESC-derived cardiovascular progenitors embedded in a fibrin patch onto the epicardium of HF patients and supported their safety (78). Our group is planning to conduct clinical trials on cardiac regenerative therapy using hPSC-CMs. Regarding post-transplantation arrhythmia, the best regimen of antiarrhythmic drugs and the best hPSC-CM status, for example, purity, maturity, and homogeneity should be further evaluated, although the mechanism and duration of arrhythmia, and the effectiveness of drugs on arrhythmia are being studied recently. We will conduct allotransplantation of hPSC-CMs whose MHC will be matched with recipient-MHC in addition to the administration of immunosuppressants. Hypoimmunogenic hPSCs have been developed through genetic modification. In the future, the clinical application of autotransplantation is expected to materialize if high-quality patient-derived hPSCs can be produced rapidly. Although further studies are required, safe and effective cardiac regenerative therapy will be realized in the near future.

## Author Contributions

YS and ST wrote the original manuscript. YM, YK, HK, KF, and ST reviewed and edited the manuscript. YS, KF, and ST acquired funding. All authors contributed to the article and approved the submitted version.

## Funding

This work was supported by Projects for Technological Development, Research Center Network for Realization of Regenerative Medicine by Japan, the Japan Agency for Medical Research and Development (AMED) grant 20bm0404023h0003 to ST, the Japan Society for the Promotion of Science (JSPS) KAKENHI 20H03768 to ST, and Grant-in-Aid for JSPS Fellows 21J10680 to YS.

## Conflict of Interest

KF is a co-founder and CEO of Heartseed, Inc. ST was an advisor of Heartseed, Inc. ST, HK, and KF owned equity in Heartseed, Inc. The remaining authors declare that the research was conducted in the absence of any commercial or financial relationships that could be construed as a potential conflict of interest.

## Publisher's Note

All claims expressed in this article are solely those of the authors and do not necessarily represent those of their affiliated organizations, or those of the publisher, the editors and the reviewers. Any product that may be evaluated in this article, or claim that may be made by its manufacturer, is not guaranteed or endorsed by the publisher.
